# Investigating the Relevance of Cyclic Adenosine Monophosphate Response Element-Binding Protein to the Wound Healing Process: An In Vivo Study Using Photobiomodulation Treatment

**DOI:** 10.3390/ijms25094838

**Published:** 2024-04-29

**Authors:** Sungyeon Kim, Jion Park, Younghoon Choi, Hongbae Jeon, Namkyu Lim

**Affiliations:** 1 Department of Plastic and Reconstructive Surgery, Dankook University College of Medicine, Cheonan 31116, Chungnam, Republic of Korea; syeon1223@dkuh.co.kr (S.K.); jeonhb110@hanmail.net (H.J.); 2 Department of Medical Laser, Graduate School, Dankook University, Cheonan 31116, Chungnam, Republic of Korea; bjo2106@gmail.com; 3 Institute of Medical Science, Dankook University Hospital, Cheonan 31116, Chungnam, Republic of Korea; chyohu@nate.com; 4 Dankook Physician Scientist Research Center (DPSRC), Dankook University Hospital, Cheonan 31116, Chungnam, Republic of Korea

**Keywords:** photobiomodulation, cAMP response element-binding protein, wound healing, inflammation

## Abstract

Monitoring inflammatory cytokines is crucial for assessing healing process and photobiomodulation (PBM) enhances wound healing. Meanwhile, cAMP response element-binding protein (CREB) is a regulator of cellular metabolism and proliferation. This study explored potential links between inflammatory cytokines and the activity of CREB in PBM-treated wounds. A total of 48 seven-week-old male SD rats were divided into four groups (wound location, skin or oral; treatment method, natural healing or PBM treatment). Wounds with a 6 mm diameter round shape were treated five times with an 808 nm laser every other day (total 60 J). The wound area was measured with a caliper and calculated using the elliptical formula. Histological analysis assessed the epidermal regeneration and collagen expression of skin and oral tissue with H&E and Masson’s trichrome staining. Pro-inflammatory (TNF-α) and anti-inflammatory (TGF-β) cytokines were quantified by RT-PCR. The ratio of phosphorylated CREB (p-CREB) to unphosphorylated CREB was identified through Western blot. PBM treatment significantly reduced the size of the wounds on day 3 and day 7, particularly in the skin wound group (*p* < 0.05 on day 3, *p* < 0.001 on day 7). The density of collagen expression was significantly higher in the PBM treatment group (in skin wound, *p* < 0.05 on day 3, *p* < 0.001 on day 7, and *p* < 0.05 on day 14; in oral wound, *p* < 0.01 on day 7). The TGF-β/TNF-α ratio and the p-CREB/CREB ratio showed a parallel trend during wound healing. Our findings suggested that the CREB has potential as a meaningful marker to track the wound healing process.

## 1. Introduction

In chronic wounds, the progression through the proliferative and remodeling stages is hindered, resulting in the wound remaining in the inflammatory phase. This prolonged inflammation is detrimental to tissue regeneration and consequently impedes the wound’s ability to heal [1,2]. A potentially effective approach is to target and rectify the underlying cellular and molecular factors that are responsible for this persistent inflammation, with the aim of restoring the wound to a healing state [3]. To gain insights into the healing state of the wound, it becomes imperative to quantify various pro-inflammatory (TNF-α, IL-1β, IL-6, etc.) and anti-inflammatory (TGF-β, IL-10, IL-13, etc.) cytokines [4,5,6,7].

Despite the wealth of well-documented pro-inflammatory and anti-inflammatory cytokines, it is worth noting that the cAMP response element-binding protein (CREB), a regulator of cellular metabolism and proliferation [8,9], has remained relatively unexplored within the realm of wound healing research. CREB is activated in response to various growth factors and inflammatory signals. Once activated, it plays a crucial role in mediating the transcription of genes that contain a cAMP-responsive element. Various immune-related cytokine genes, including IL-2, IL-6, and TNF-α, possess this cAMP-responsive element. Furthermore, phosphorylated CREB has been suggested to have a direct inhibitory effect on NF-κB activation. It achieves this by obstructing the binding of the CREB to the NF-κB complex, effectively curbing proinflammatory responses [10,11]. This multifaceted role of CREB underscores its potential significance in the regulation of inflammatory processes, which could have implications in wound healing research and therapies.

Meanwhile, photobiomodulation (PBM) is a therapeutic technique that harnesses the power of red or near-infrared-wavelength light to stimulate and enhance the function of the living body [12,13]. It improves wound healing by inducing increases in mitotic activity, in the numbers of fibroblasts, in collagen synthesis, and in neovascularization in a non-invasive manner. Although at a lower penetration depth, blue or green light also promotes wound healing and reduces inflammation [14,15]. Consequently, PBM treatment has been recognized for its efficacy in facilitating the healing process of both acute and chronic wounds, as well as its ability to inhibit the formation of scar tissue [16,17].

Thus, our objective is to analyze the cytokines that undergo changes throughout the various stages of wound healing under photobiomodulation treatment. Moreover, we intend to investigate the potential correlations between these cytokine changes and alterations in CREB activity.

## 2. Results

### 2.1. The PBM Treatment Accelerated Wound Healing

**Figure 1:**
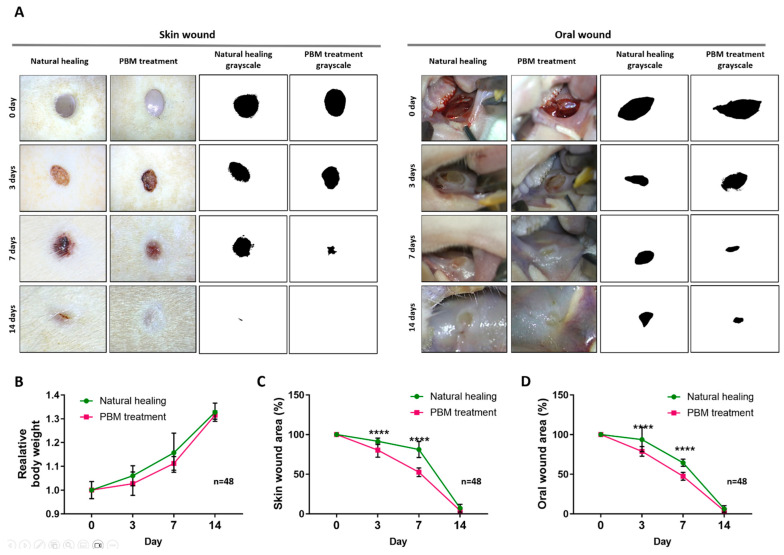
Photograph of oral and skin punch biopsy wounds healing on 0, 3, 7, and 14 days. (**A**) Morphological representation of oral and skin biopsy punch wound showing during wound healing. (**B**,**C**) Graph of percent of wound area on oral mucous membrane and skin. Each point represents the mean percentage of wound area. On days 3 and 7, the PBM treatment group’s percent of wound closure was significantly lower than the natural wound healing group in both oral and skin wounds. Data are shown as the mean ± SEM. (**D**) Relative body weight during wound healing. There is no significant difference in body weight. **** *p* < 0.0001.

Through the 14-day healing tracing, the PBM treatment significantly reduced the wound size of the oral mucous membrane and skin on days 3 and 7 compared to the NH group [relative wound size, NH vs. PBM treatment groups in oral wound: 93.7 ± 15.2% vs. 78.8 ± 6.1% (day 3, *p* < 0.0001) and 64.5 ± 4.6% vs. 47.7 ± 4.9% (day 7, *p* < 0.0001); relative wound size, NH vs. PBM treatment groups in skin wound: 91.5 ± 4.1% vs. 80.4 ± 8.9% (day 3, *p* < 0.0001) and 81.2 ± 10.1% vs. 52.5 ± 5.4% (day 7, *p* < 0.0001)] (Figure 1A–C, Table 1). Compared with skin wounds, oral wounds that were treated with PBM showed accelerated wound closure without complications. Meanwhile, there was no significant difference in the body weights among the groups for 14 days (Figure 1D).

### 2.2. Histological Analysis

**Figure 2:**
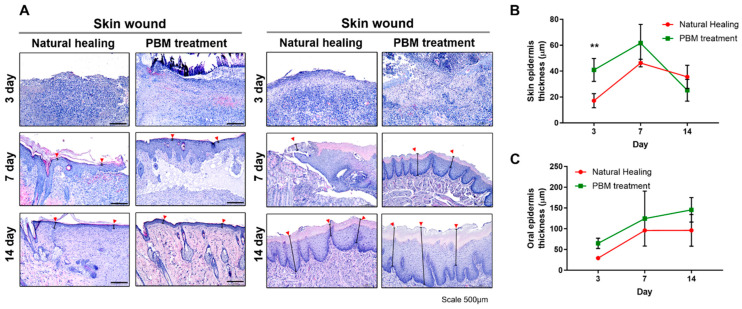
Histopathological analysis of the oral and skin wound tissue during wound healing. (**A**) H&E staining of oral and skin tissues surrounding punch biopsy wound region. PBM treatment promotes oral and skin wound healing. (**B**,**C**) Quantification analysis of the thickness of the skin and oral sound region epidermis. On day 3 after injury, the epidermis of the skin wound treated with PBM was significantly thicker than the natural healing tissue. Data are shown as the mean ± SEM. ** *p* < 0.01. Scale bar = 500 μm. Red arrow: Epidermal thickness.

**Figure 3:**
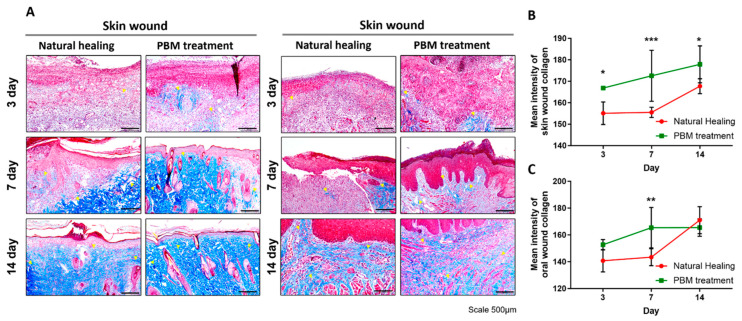
Masson-trichrome staining showed the distribution and density of collagen in tissue during wound healing. (**A**) Masson-trichrome (MT) staining of oral and skin wounds at 3, 7, and 14 days. Collagen was stained blue. The PBM treatment group showed a more collagen-positive region compared to the natural healing group. Scale bar = 100 μm. (**B**,**C**) Quantification analysis of the mean intensity of collagen. The PBM treatment group’s collagen intensity was significantly higher than that of the natural healing group. Yellow arrows indicate collagen components. Data are shown as the mean ± SEM. * *p* < 0.05, ** *p* < 0.01, *** *p* < 0.001.

In the H&E staining analysis, on day 3 within the skin area, it was evident that the NH group did not exhibit distinct epidermal formation, whereas the PBM treatment group displayed a significant onset of epidermal regeneration (epidermal thickness, which calculated the mean of three points; the shortest, middle, and longest, 17.2 ± 5.5 μm in NH group vs. 40.9 ± 8.9 μm in PBM treatment group, *p* = 0.0032) (Table 1). By day 7, no significant difference between the two groups was observed, although there was a trend towards a thicker epidermis in the PBM treatment group compared with that in the NH group. However, after 14 days, when both groups achieved complete epidermal healing, there was no statistically significant difference in the final epidermal thickness (Figure 2A,B, Table 1). Meanwhile, within the oral region, both the NH and PBM treatment groups demonstrated a progressive increase in epidermal thickness over the healing period, but no statistically significant difference was observed between these groups throughout the entirety of the healing process (Figure 2A,C, Table 1). In Masson-trichrome staining, except for the oral area on days 3 and 14, it was consistently observed that collagen expression was significantly elevated in the PBM treatment group compared with that in the NH group, irrespective of the wound location, throughout the entire duration of the experiment [collagen deposition area, NH vs. PBM treatment groups in oral wound: 143.4 ± 6.3 μm^2^ vs. 165.4 ± 15.1 μm^2^ (day 7, *p* = 0.0020); collagen deposition area, NH vs. PBM treatment groups in skin wound: 155.1 ± 5.2 μm^2^ vs. 166.9 ± 0.3 μm^2^ (day 3, *p* = 0.0167), 155.5 ± 2.4 μm^2^ vs. 172.6 ± 11.9 μm^2^ (day 7, *p* = 0.0007) and 167.7 ± 3.5 μm^2^ vs. 177.9 ± 8.6 μm^2^ (day 14, *p* = 0.0260)] (Figure 3, Table 1). 

### 2.3. Tracing the Progress of RNA and Protein Change during Wound Healing

**Figure 4:**
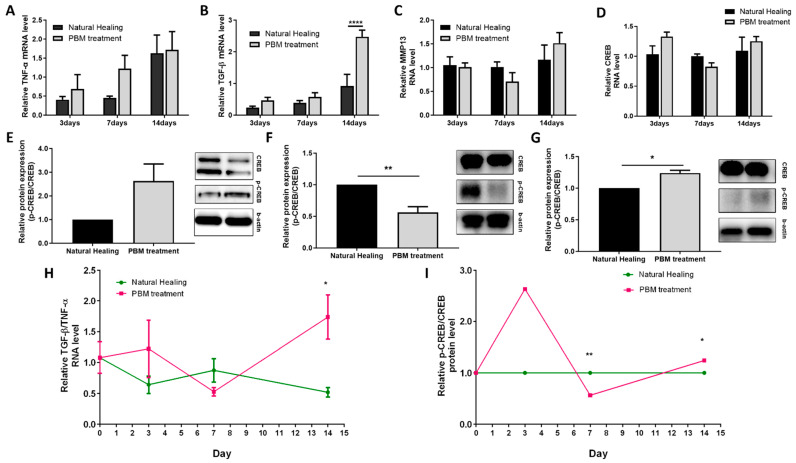
Analysis of the level of TNF-α, TGF-β, MMP 13, and CREB during wound healing. (**A**–**D**) The relative RNA level of TNF-α, TGF-β, MMP13, and CREB at 3, 7, and 14 days. (**E**) Protein expression of p-CREB and CREB evaluated by Western blot and relative protein expression level of p-CREB and CREB ratio on day 3. (**F**) The results of Western blot on day 7. (**G**) The results of Western blot on day 14. (**H**) The ratio of TGF-β and TNF-α RNA level. (**I**) The ratio of p-CREB and CREB protein levels. All data are shown as the mean ± SEM. * *p* < 0.05, ** *p* < 0.01, **** *p* < 0.0001.

Both TNF-α and TGF-β cytokines exhibited a gradual trend of increasing mRNA levels extending up to day 14, in both the PBM treatment and NH groups. Notably, by day 14, TGF-β displayed a significantly higher expression in the PBM treatment group (relative mRNA level, 0.9 ± 0.7 in NH group vs. 2.5 ± 0.4 in PBM treatment group, *p* < 0.0001) (Figure 4A,B, Table 1). And the TGF-β/TNF-α ratio showed a fluctuation pattern that was higher in the PBM treatment group compared to the NH group on day 3, experienced a decline on day 7, and then was significantly higher on day 14 (relative mRNA level, 0.5 ± 0.1 in NH group vs. 1.7 ± 0.4 in PBM treatment group, *p* = 0.0116) (Figure 4H, Table 1). Meanwhile, the expression of MMP13, a marker primarily implicated in processes associated with the remodeling phase, did not manifest a significant difference between the two groups by the conclusion of day 14 (Figure 4C, Table 1). Similarly, the analysis of RNA expression pertaining to CREB, a novel analytical marker, did not reveal a statistically significant difference between the two groups within the same timeframe (Figure 4D, Table 1). 

To identify the extent of CREB activation, we calculated the ratio of p-CREB to unphosphorylated CREB. Intriguingly, this ratio exhibited a temporal pattern that closely mirrored the fluctuations that were observed in the timeline of wound healing progression, as indicated by the TGF-β/TNF-α ratio. On day 3, the PBM treatment group displayed a tendency for a higher p-CREB/CREB ratio, which declined by day 7 (folded protein level, NH vs. PBM treatment groups; 1 vs. 0.6 ± 0.1, *p* < 0.0081), and then exhibited a significant increase by day 14 (folded protein level, NH vs. PBM treatment groups; 1 vs. 1.2 ± 0.04, *p* < 0.0289) (Figure 4E–G,I, Table 1).

## 3. Discussion

The oral environment has a pronounced upregulation of cytokines, which stimulate epidermal proliferation and migration. This demonstrates a remarkable capacity for rapid wound healing following traumatic injury. Moreover, saliva in the oral cavity contains elevated levels of lysozyme and hyaluronic acid, contributing to the establishment of a humid microenvironment that is characterized by potent antibacterial properties, thereby facilitating the process of wound healing [18,19]. Our hypothesis posited that wound healing would exhibit variations not only in response to PBM treatment but also in relation to the wound’s location (oral vs. skin). However, upon conducting an evaluation of the histological analyses, which included macroscopic observations, assessments of epidermal thickness, and examinations of collagen content, it became evident that the presence or absence of PBM treatment played a more pivotal role in influencing wound healing than the inherent tissue distinctions between the wound locations. Notably, the discernible contrast between the PBM-treated and NH groups within the skin region was particularly prominent.

The pace of tissue reconstruction in structures is profoundly influenced by the expression of cytokines and other anti-inflammatory factors that govern the processes of cell proliferation, differentiation, and migration, regardless of wound location [19,20,21]. And the most crucial factor in wound healing is the prompt entry into the proliferative phase. To validate this phase, we conducted an analysis focusing on TNF-α and TGF-β, two of the most prominent pro-inflammatory and anti-inflammatory cytokines, respectively [20,22,23]. We observed the transition from inflammatory to proliferative phases, as quantified by the TGF-β/TNF-α ratio. The proliferative phase, conventionally overlapping with the inflammatory phase and peaking after approximately one week [24], appeared to commence marginally earlier in PBM treatment group. This deviation may be attributed to the more robust induction of anti-inflammatory reactions facilitated by PBM treatment. In detail, it revealed heightened ratios in the PBM treatment group on day 3 and in the NH group on day 7. These fluctuations in ratio closely parallel the visually observed wound size outcomes. Until day 7, substantial tissue reduction was prominent in the PBM treatment group, after which the NH group exhibited a more pronounced rate of wound reduction compared to the PBM treatment group. 

Meanwhile, both NH and PBM treatment groups exhibited an increasing trend in the relative values of TGF-β and TNF-α until day 14. Especially, the elevation of TGF-β on the 14th day within the PBM treatment group demonstrated a significant difference from the NH group. These findings contrast with the outcomes of a study conducted by Houreld NN et al., wherein a reduction in TNF-α was observed in diabetic wounded fibroblast cells subjected to 830 nm light [25]. The attenuation of pro-inflammatory cytokines following PBM treatment is well-known knowledge, substantiated by various references [25,26,27,28,29,30]. However, our result is similar to a study by Ahmed OM et al., which investigated skin wounds in diabetic rats using 632.8 nm light. In that study, serum TNF-α levels on day 14 manifested an increase in most groups, with variations ranging from approximately 0.95 to 2.42 times contingent upon the detailed experimental group [31]. An elevation tendency of both pro- and anti-inflammatory cytokines was comprehended from histological findings, where an abundance of inflammatory cells persisted in the day 14 histological images. It was assumed that the proinflammatory reaction may have continued due to the presence of residual wound tissue despite the decrease in wound size over the two-week period. 

This phenomenon also offered an explanation for the observed lack of significant changes in MMP13 expression across all groups during the two-week period. MMP13, which is known to be highly active during the remodeling phase of wound healing [32], might not have exhibited substantial alterations, because the wound healing process appeared to have been influenced by ongoing inflammation and possibly additional pro-inflammatory and anti-inflammatory responses induced by the PBM treatment. As a result, the typical patterns of MMP13 expression that are associated with wound remodeling may have been obscured by these concurrent inflammatory processes. Similarly, CREB did not demonstrate significant alterations in the RNA analysis. The absence of substantial alterations in CREB expression was consistent with the characteristic mode of CREB activation, which predominantly takes place via phosphorylation [11,33]. In other words, CREB RNA levels may not exhibit substantial variations regardless of phosphorylation. To comprehensively analyze CREB, it was more suitable to investigate the protein expression, as this allowed for the assessment of the CREB phosphorylation status.

CREB is recognized for its pivotal role in various cellular processes in neurons, including proliferation, differentiation, survival, long-term synaptic potentiation, neurogenesis, and neuronal plasticity [34,35]. Studies have also demonstrated the activation of CREB in response to muscle tissue damage, where it contributes to muscle regeneration [31]. While there have been numerous studies exploring the relationship between CREB and central nervous system diseases, such as dementia and schizophrenia [36,37], there has been relatively limited research on CREB in the context of wound healing. In this study, the analysis focused on monitoring the changes in both p-CREB and unphosphorylated CREB during the wound-healing process. Notably, the alterations that were observed in p-CREB and CREB paralleled the trends that were observed for TGF-β and TNF-α. These outcomes implied that the progression of wound healing can potentially be assessed and inferred through the analysis of a single factor, CREB, as opposed to relying on a combination of various cytokines. 

Meanwhile, the CREB-binding protein (CREBBP) and its paralog p300 function as lysine acetyl transferases (KAT) within the KAT3 protein family, specializing in histone modification to regulate chromatin accessibility and transcription. CBP and p300 are recognized as tumor suppressor genes due to their role in acetylating p53, a key guardian of genome stability [38]. Furthermore, they potentially enhance DNA repair processes by means of histone acetylation, activating transcription and aiding in the recruitment of DNA repair factors to the damaged site [38,39]. Eventually, when CREB is phosphorylated, it contributes to the activation of CBP/p300, inducing changes at the DNA level [40]. Although this factor was not explored in this study, it will be crucial to investigate CBP/p300 when analyzing the in-depth mechanism of CREB in wound healing in a further study. Therefore, this matter is one of limitations to this study. Furthermore, there are additional limitations to our research, as follows. To comprehensively assess the wound healing process including macrophage polarization, simultaneous analysis of multiple cytokines would be more rational (IL-1 β, IL-6, CD86, iNOS, etc., for M1 detection; IL-4, IL-10, CD206, Arg-1, etc., for M2 detection) [41,42]. However, in our study, we only examined one representative cytokine at a time. Lastly, it is anticipated that the quality of residual scars may vary based on the differences in collagen deposition due to PBM treatment. However, in this study, a more in-depth investigation into scars was not conducted. So, additional follow-up studies are deemed necessary to address this aspect. 

## 4. Materials and Methods 

### 4.1. Skin or Oral Wound Animal Model and Groups

**Figure 5:**
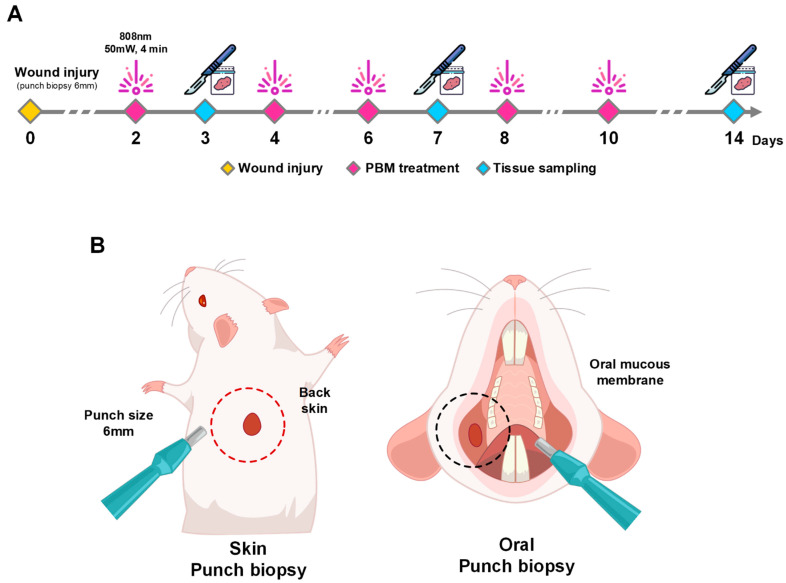
Scheme of working protocol and animal model. (**A**) Schedule of PBM treatment and tissue sampling. Wound tissues were sampled at 3, 7, and 14 days after punch biopsy. Wound tissue was treated by PBM at 50 mW, 4 min, and 5 times during wound healing. (**B**) Location of wounds. Surgical punch biopsies 6 mm in diameter were used to make wounds. The wounds were located on the oral mucous membrane and back skin.

This study was conducted according to the guidelines by the Institutional Animal Care and Use Committee at Dankook University (DKU-20-057). A total of 48 seven-week-old male Sprague Dawley (SD) rats (Orientbio Inc., Sungnam, Republic of Korea) were used as subjects, and all animals were housed in temperature- and light-controlled rooms (12/12 dark/light cycle) for 3 days before surgery. Rat dorsal skin or buccal oral mucosa was removed to create a wound using a 6 mm punch biopsy device (KAI medical Inc., Seki-shi, Japan) while the rats were under sedation that was induced by Zoletil (Virba Animal Health, Seoul, Republic of Korea) at a dosage of 15 mg/kg (Figure 5). Four groups were established based on wound location (skin vs. oral) and healing method (natural vs. PBM treatment): (1) natural healing (NH) in the skin wound group, (2) PBM treatment in the skin wound group, (3) NH in the oral wound group, and (4) PBM treatment in the oral wound group. This study involved the sacrifice of four animals at three different time points, on days 3, 7, and 14, following the wound creation, to facilitate subsequent analysis (Figure 5).

### 4.2. Photobiomodulation (PBM) Treatment

In Groups B and D, a laser with a wavelength of 808 nm (WELS01; WelsMeditech Co., Cheonan, Republic of Korea) was utilized according to the manufacturer’s instructions. The laser settings were adjusted to a power density of 63.69 mW/cm^2^. The laser power was measured using a power meter (PD300-TP-ROHS, Ophir Optronics, Jerusalem, Israel). To attain the specified energy exposure, each group underwent five sessions of laser shots; the duration of each session was 240 s. The specific parameters used for PBM treatment are found in Table 2. Laser treatment sessions were conducted every other day over an 8-day period (on days 2, 4, 6, 8, and 10 after wound creation) under anesthesia, totaling 60 J of energy exposure per animal. The selection of this energy value was based on prior research indicating which is most effective for promoting wound healing [5]. Throughout the treatment period, the weights of the animals and the sizes of the wounds were recorded for monitoring and assessment (Figure 5). The wound size was assessed with a caliper, and its area was computed using the elliptical formula: A = D (long distance) × d (short distance) × π/4.

### 4.3. Histological Analysis

#### 4.3.1. Tissue Preparation

For tissue sample collection at the specified time points (3, 7, and 14 days after surgery), an 8 mm diameter punch biopsy device (KAI Medical) was used. The harvested tissue samples were then preserved by fixing them in a 10% neutral buffered formalin solution. Subsequently, the fixed tissue samples were embedded in paraffin, sectioned (5 μm thickness), and mounted onto slides for further analysis and examination.

#### 4.3.2. Hematoxylin and Eosin Staining

The slides underwent a deparaffinization and rehydration process within a staining jar. This process included sequential immersion in xylene for 5 min, followed by a series of ethanol solutions with concentrations of 100%, 95%, 95%, 90%, and 80%, each for a duration of 3 min. Subsequently, the slides were rinsed with tap water for 2 min. Following the rehydration steps, the slides were subjected to staining using Harris Hematoxylin (Sigma-Aldrich Co., St. Louis, MO, USA) for a duration of 2 min, after which they were rinsed again with tap water. To remove excess color, the slides were briefly immersed in 1% acid alcohol and rinsed once more in tap water. Following this, the slides were stained with Eosin (Sigma-Aldrich) for 2 min. After the staining process was completed, the slides were immersed successively in ethanol and xylene and then mounted with a coverslip, using DPX mounting medium (Sigma-Aldrich). The epidermal thickness within the wound region was calculated using the mean value of three points (longest, shortest, and mid-point), and subsequently quantified using Image J software version 1.53e (National institutes of Health [NIH], Bethesda, MD, USA). 

#### 4.3.3. Collagen Staining in Masson’s Trichrome Staining

Similar to the process of H&E staining, the slides were subjected to deparaffinization and rehydration by immersion in xylene followed by a graded series of ethanol solutions. Subsequently, the tissue slides were placed in Bouin’s solution (Sigma-Aldrich) at 60 °C for 45 min. For cytoplasm staining, acid fuchsin (Sigma-Aldrich) staining was applied for a duration of 5 min. Afterward, the slides were rinsed in tap water. Following this step, the slides were exposed to a phosphomolybdic acid solution (Sigma-Aldrich) for 10 min, followed by staining with a methyl blue solution (Sigma-Aldrich) to highlight collagen. Once the staining procedure was completed, the slides underwent a dehydration process involving a series of ethanol solutions. Finally, they were mounted with a coverslip using DPX mounting medium (Sigma-Aldrich). Quantification of the collagen fibers was counted with software of Image J (NIH).

### 4.4. Real-Time Polymerase Chain Reaction (RT-PCR)

Total RNA was extracted using RiboEX (GeneAll, Seoul, Republic of Korea). Before cDNA synthesis, the RNA concentrations were measured using a NanoDrop spectrophotometer (ND-1000; NanoDrop, Wilmington, DE, USA); 1 µg of total RNA was reverse-transcribed using Hyperscript^TM^ 2X RT Master mix (GeneAll). qRT-PCR was performed using AccuPower^®^ 2× GreenStar™ qPCR Master Mix (Bioneer, Daejeon, Republic of Korea) and gene-specific primers (Table 3) in an RT-PCR system (ABI 7500; Applied Biosystems, Foster City, CA, USA). Each target gene expression level was normalized to endogenous GAPDH using the formula [ΔCt = Ct (target gene) − Ct (GAPDH)]. The 2^−ΔΔCt^ method was applied to calculate the relative quantification value of target genes to control samples.

### 4.5. Western Blot

The wound tissue was collected through punching and homogenized in RIPA buffer (Sigma-Aldrich) with 1 mg/mL of a protease inhibitor and phosphatase inhibitor cocktail. The homogenized samples were centrifuged at 15,000 rpm for 15 min at 4 °C, and supernatants were collected. The Bicinchoninic acid assays (Thermo Fisher Scientific Inc., Waltham, MA, USA) were performed to determine the protein concentration of the supernatants. Anti-CREB (#9197, 1:1000; Cell Signaling Technology, Beverly, MA, USA) and anti-phospho-CREB (#9198, 1:1000; Cell Signaling Technology) were used to detect the CREB and phosphorylated CREB (p-CREB) proteins. Horseradish peroxidase-conjugated goat anti-rabbit IgG antibodies and goat anti-mouse IgG antibodies were used as secondary antibodies (all antibodies from Thermo Fisher Scientific). Protein band intensity was analyzed using Image J software (NIH). CREB and p-CREB protein levels were normalized to the β-actin (Cell Signaling Technology).

### 4.6. Statistical Analysis

All data between the NH and PBM treatment groups are expressed as the mean ± SEM and were analyzed using a *t*-test, where *p* < 0.05 was considered statistically significant. GraphPad Prism 6.02 software (GraphPad Software Inc., La Jolla, CA, USA) was used for statistical analysis.

## 5. Conclusions

Through serial analysis of the wound healing process, our findings suggested that the CREB factor holds strong potential as a meaningful marker for tracking the progression of wound healing.

## Figures and Tables

**Table 1:** The numerical values of results.

Relative Wound Size (%)		3 Days	7 Days	14 Days
Oral	Natural healing	93.7 ± 15.2	64.5 ± 4.6	6.1 ± 4.1
	PBM treatment	78.8 ± 6.1	47.7 ± 4.9	3.9 ± 3.1
*p* value		**<0.0001**	**<0.0001**	0.9438
Skin	Natural healing	91.5 ± 4.1	81.2 ± 10.1	7.1 ± 4.9
	PBM treatment	80.4 ± 8.9	52.5 ± 5.4	4.0 ± 3.7
*p* value		**<0.0001**	**<0.0001**	0.2443
**Epidermis thickness** (μm)		**3 days**	**7 days**	**14 days**
Oral	Natural healing	29.2 ± 3.2	95.8 ± 12.9	96.3 ± 38.1
	PBM treatment	64.8 ± 12.4	124.4 ± 66.1	145.6 ± 29.5
*p* value		0.8627	0.8134	0.1249
Skin	Natural healing	17.2 ± 5.5	46.3 ± 2.9	35.6 ± 8.9
	PBM treatment	40.9 ± 8.9	61.6 ± 14.5	25.2 ± 8.4
*p* value		**0.0032**	0.1727	0.1384
**Collagen deposition area** (μm^2^)		**3 days**	**7 days**	**14 days**
Oral	Natural healing	140.8 ± 8.3	143.4 ± 6.3	171.0 ± 10.1
	PBM treatment	152.7 ± 3.9	165.4 ± 15.1	165.5 ± 6.5
*p* value		0.3408	**0.0020**	0.7096
Skin	Natural healing	155.1 ± 5.2	155.5 ± 2.4	167.7 ± 3.5
	PBM treatment	166.9 ± 0.3	172.6 ± 11.9	177.9 ± 8.6
*p* value		**0.0167**	**0.0007**	**0.0260**
**Relative mRNA level**		**3 days**	**7 days**	**14 days**
TNF-α	Natural healing	0.4 ± 0.2	0.5 ± 0.1	1.6 ± 1.0
	PBM treatment	0.7 ± 0.8	1.2 ± 0.7	1.7 ± 1.0
*p* value		0.9246	0.3604	0.9975
TGF-β	Natural healing	0.2 ± 0.1	0.4 ± 0.1	0.9 ± 0.7
	PBM treatment	0.5 ± 0.2	0.6 ± 0.3	2.5 ± 0.4
*p* value		0.7913	0.8580	**<0.0001**
TGF-β /TNF-α	Natural healing	0.6 ± 0.1	0.9 ± 0.2	0.5 ± 0.1
	PBM treatment	1.2 ± 0.5	0.5 ± 0.1	1.7 ± 0.4
*p* value		0.4193	0.8278	**0.0116**
MMP13	Natural healing	1.1 ± 0.3	1.0 ± 0.2	1.2 ± 0.6
	PBM treatment	1.0 ± 0.2	0.7 ± 0.4	1.5 ± 0.4
*p* value		0.9982	0.6260	0.5314
CREB	Natural healing	1.0 ± 0.3	1.0 ± 0.1	1.1 ± 0.5
	PBM treatment	1.3 ± 0.1	0.8 ± 0.1	1.3 ± 0.2
*p* value		0.2789	0.6801	0.7441
**Folded protein level** **(p-CREB/CREB)**		**3 days**	**7 days**	**14 days**
p-CREB /CREB	Natural healing	1	1	1
	PBM treatment	2.6 ± 0.7	0.6 ± 0.1	1.2 ± 0.04
*p* value		0.0638	**0.0081**	**0.0289**

PBM, photobiomodulation; TNF, tumor necrosis factor; TGF, transforming growth factor; MP, matrix metalloproteinase; CREB, cAMP response element binding protein. All parameters are shown as mean ± standard error.

**Table 2:** Detail parameters of photobiomodulation treatment in this study.

Parameter	Value
Wavelength (nm)	808
Power output (mW)	50
Energy output (J/s)	0.05
Energy density (mW/cm^2^)	63.69
Spot size (mm)	10
Frequency (Hz)	10
Pulse duration (ms)	1
Duration of irradiation (s)	240
Pulse energy per session (J)	12
Pulse energy density per session (mW/cm^2^)	15.28
Number of sessions	5
Total irradiated energy (J)	60
Total energy density (J/cm^2^)	76.4

**Table 3:** Primer sequence for the real-time polymerase chain reaction.

Gene	Forward Primers	Reverse Primers
TNF-α	GACCCTCACACTCAGATCATCTTCT	CGTAGCCCACGTCGTAGCA
TGF-β	AGGGCTACCATGCCAACTTC	CCACGTAGTAGACGATGGGC
MMP13	ACCATCCTGTGACTCTTGCG	TTCACCCACATCAGGCACT
CREB	AGCTGCCACTCAGCCGGGTA	TGGTGCTAGTGGGTGCTGTG

## Data Availability

The authors confirm that the data supporting the findings of this study are available within the article. Raw data that support the findings of this study are available from the corresponding author upon reasonable request.
